# Supplementary material for: Cobalt Protoporphyrin Downregulates Hyperglycemia-Induced Inflammation and Enhances Mitochondrial Respiration in Retinal Pigment Epithelial Cells
Source: Antioxidants (Basel). 2025 Jan 15;14(1):92. doi: 10.3390/antiox14010092 (PMC11759195; doi:10.3390/antiox14010092)
Supplement: Supplementary file 1 [file antioxidants-14-00092-s001.zip › antioxidants-3377321-supplementary.pdf]

**Table S1. Lists of primary and secondary antibodies were used for the Western blotting analysis in the present study.**

| Name of Antibodies | Company | Catalog Number |
|---|---|---|
| Heme Oxygenase 1 | Elabscience | E-AB-93300 |
| IL1 beta | Genetex | GTX130021 |
| TNF alpha | Genetex | GTX110520 |
| Activated Caspase 3 | Arigo biolaboratories | ARG57512 |
| VEGF | Genetex | GTX20119 |
| PGC1 alpha | Invitrogen | PA5-72948 |
| mtTFA | Genetex | GTX112760 |
| Nrf1 | Genetex | GTX103179 |
| Ki67 | Genetex | GTX16667 |
| TGF beta 1 | Genetex | GTX130023 |
| Nrf2 | Genetex | GTX103322 |
| Beta-actin | Genetex | GTX109639 |
| Goat Anti-Rabbit IgG | Jackson ImmunoResearch | 111-035-144 |
| Goat Anti-Mouse IgG | Jackson ImmunoResearch | 115-035-003 |
